# Effect of the Synthetic Approach on the Formation and Magnetic Properties of Iron-Based Nanophase in Branched Polyester Polyol Matrix

**DOI:** 10.3390/ijms232314764

**Published:** 2022-11-25

**Authors:** Artur Khannanov, Anastasia Burmatova, Klara Ignatyeva, Farit Vagizov, Airat Kiiamov, Dmitrii Tayurskii, Mikhail Cherosov, Alexander Gerasimov, Evtugyn Vladimir, Marianna Kutyreva

**Affiliations:** 1 Butlerov Chemistry Institute, Kazan Federal University, 420008 Kazan, Russia; 2 Institute of Physics, Kazan Federal University, 420008 Kazan, Russia; 3 Interdisciplinary Center “Analytical Microscopy”, Kazan Federal University, 420008 Kazan, Russia

**Keywords:** hyperbranched polyesters, zero-valent nanoparticles, iron oxide nanoparticles, synthetic approach

## Abstract

This article shows the success of using the chemical reduction method, the polyol thermolytic process, the sonochemistry method, and the hybrid sonochemistry/polyol process method to design iron-based magnetically active composite nanomaterials in a hyperbranched polyester polyol matrix. Four samples were obtained and characterized by transmission and scanning electron microscopy, infrared spectroscopy and thermogravimetry. In all cases, the hyperbranched polymer is an excellent stabilizer of the iron and iron oxides nanophase. In addition, during the thermolytic process and hybrid method, the branched polyol exhibits the properties of a good reducing agent. The use of various approaches to the synthesis of iron nanoparticles in a branched polyester polyol matrix makes it possible to control the composition, geometry, dispersity, and size of the iron-based nanophase and to create new promising materials with colloidal stability, low hemolytic activity, and good magnetic properties. The NMR relaxation method proved the possibility of using the obtained composites as tomographic probes.

## 1. Introduction

The design, the study of the properties, and the practical application of metal–polymer composites are some of the topical areas of advanced materials science [1,2,3,4,5]. This is due to the variety of their potential applications, such as for catalysis [6,7], water purification [8], energy storage, and conversion elements [9,10,11]; the list of applications is endless. Among the application areas of metal-containing nanocomposites, biomedicine can be singled out as the most important [12,13,14]. Combining the controllable properties and architecture of synthetic polymers with the functional properties (e.g., magnetic activity, luminescence, and bioactivity) of metal nanoparticles or their binary compounds makes it possible to produce unique smart materials for nanotherapeutics, diagnostics, and theranostics. Composite magnetoactive smart nanomaterials based on iron and its oxides are a significant part of these studies.

There are many applications of iron/iron oxide in biomedicine, but three main ones can be distinguished. The first comprises the sensitive sensors and bioimaging agents for the early detection of diseases [15]. Major developments are underway to advance and discover imaging agents for MRI techniques [16,17,18]. For direct optical imaging, the conjugation of luminescent magnetoactive nanoparticles with target disease markers is used [19,20]. It is also possible to combine the imaging and therapeutic components, such as in the use of nanoparticles as radioactive labels, including, for example, Fe_3_O_4_ oxide nanoparticles containing ^59^Fe [21,22].

Secondly, there is the creation of targeted drug delivery systems based on them [23,24]. The advantage of drug delivery systems based on magnetically susceptible materials is the controlled targeting of nanocontainers for the formation of conjugates with tumor-specific antibodies, which contributes to the selective accumulation of nanoparticles and the suppression of the disease focus [25,26,27,28]. A number of studies have shown that the presence of an oxide shell makes it easy to modify the surface of nanoparticles, with, for example, the use of Herceptin, which is an antibody for the HER2/neu protein, to give them additional vector properties for certain tissues [29].

Thirdly, there are nanoparticles used as theranostic agents [30], and there are physical therapeutic systems, such as those with nanoparticles for magnetic hyperthermia [31]. By themselves, biophilic metal nanoparticles have their own background therapeutic activity and adjustable specific activity against opportunistic infections and viruses that are no less dangerous than those of oncology [32,33]. The ability to functionalize the surface of particles and simultaneously control their behavior using an external magnetic field makes them an indispensable material for theranostics—a technology for combining therapeutic effects and diagnostics in one agent [34]. In [35], the authors demonstrate the possibility of using Fe_3_O_4_@Au nanoparticles as an MRI contrast agent and antibody carrier for targeted delivery to a prostate tumor. 

Among the three areas identified, the most actively developing research is in the creation of iron nanoparticle-based chemo-phototherapy systems for cancer treatment [36]. Malignant tumors of the endocrine system develop in the endocrine glands; so, cancer cells quickly enter the blood and lymph system. Tumors of the thyroid gland, ovaries, and adrenal glands are the most common and aggressive forms of the endocrine system cancers. All of them lead to hormonal imbalance and severe metabolic disorders. Currently, as part of the complex therapy for cancer of the endocrine system, the possibilities of the method of local hyperthermia of tumors using magnetic fields are shown [37,38,39,40]. The structure and composition of the composite particles based on nano-Fe_3_O_4_ are the basis for the successful application of this method.

The nature of the nanoparticle stabilizer is a key factor in controlling the properties of these systems and their subsequent biomedical applications. Silicate nanoparticles [41], zeolites [42], various sugars [43,44], graphene oxide [45,46,47,48,49], and surfactants [50,51] are often used as matrices for the stabilization and preparation of metal nanoparticles. A nanoparticle stabilizer used for the synthesis of magnetic nanocomposites based on iron oxides for biomedical applications should have three main characteristics: firstly, to reduce interparticle interactions, it is necessary to use magnetically inactive polymer matrices; secondly, the polymer must be biocompatible, biosimilar, and biodegradable; and thirdly, its composition should be predictable and constant. [52,53].

The use of synthetic branched polymers [54,55] for the synthesis of metal or metal oxide nanocomposites makes it possible to combine the properties of a metal nanophase with the individual reological and mechanical properties of polymers [49,56,57]. Branched polymers have a well-established chemistry for their synthesis and offer many ways to customize their functionality. The core-shell architecture ensures their operation as macromolecular “nanoreactors” and nanoporous stabilizers in the synthesis of metal-containing nanoparticles [58,59,60]. The multimodal capabilities of branched polymers, combined with their easy and controlled synthetic engineering, have resulted in properties that are ideal for their use as stabilizers and supramolecular carriers of metal nanoparticles, allowing them to control their morphology and ultimately control the properties of materials. 

At the same time, most of the works are devoted to the use of dendrimers, namely polyamidomine (PAMAM) or propyleneimine for the synthesis of the magnetic NPs of iron compounds [61,62,63,64,65,66,67,68,69]. Among a small number of examples of the use of hyperbranched polymers to stabilize Fe_3_O_4_ nanoparticles are branched polyamines HAPAM [70], HBPAMAM [71], PAMAM-b-PEG-FA [72], and PEI [73]. However, the presence of the NH fragment in the molecule leads to the high cytotoxicity of PAMAM and the hyperbranched polyamines [45]. Much less commonly used are the hyperbranched polyglycerols [74,75] and polyesters [76]. At the same time, their stabilizing ability and accessibility for terminal modification are not inferior to those of branched polyamines, and their toxicity is much lower [77]. We want to draw attention to the fact that in almost all the studies, Fe_3_O_4_ nanoparticles are a structural element of a polymer-composite nanomaterial and are used primarily as a magnetoactive component in a general application.

A recent good review by Lee et al. [78] detailed the size/geometry–property relationship of Fe_3_O_4_ nanoparticles; the size/geometry–application relationship; and the role of nanostructures in targeted applications. Data are presented on the Fe-nanophase obtained by various methods (thermal decomposition/solvothermal/hydrothermal/sonochemistry, etc.) and under conditions of stabilization by compounds different in nature and chemical composition. Our early papers present the results of a scientific group studying the influence of the architecture of a stabilizer polymer; the process of the preorganization of metal ions on a polymer matrix; and the morphology, magnetic activity, and biological properties of hyperbranched polyester polyols doped with cobalt nanoparticles/cobalt oxides [79,80,81]. A number of similar studies, but for iron nanoparticles in a PCA-PEG HBP matrix, were carried out by Niknejad et al. [82] and Khann et al. [83].

An analysis of the works on this indicator shows that the characteristics of the nanophase morphology represent the most complete way to obtain the nanoparticles of magnetite and a natural stabilizer. At the same time, it is no less important to understand the processes of the formation of the Fe_3_O_4_ nanophase within the framework of one system, the precursor of the iron nanoparticles and the stabilizer, depending on the synthetic approach used. This will make it possible to optimize the synthetic process for obtaining magnetically active polymer-stabilized Fe_3_O_4_ nanoparticles, taking into account their individual target properties and the functional characteristics of the nanomaterial.

That is why the present work was aimed at revealing the relationship between the synthesis approach and the chemical composition, morphology, and magnetic activity of the iron oxides nanophase in a hyperbranched polymer matrix.

## 2. Results

This section may be divided by subheadings. It should provide a concise and precise description of the experimental results, their interpretation, as well as the experimental conclusions that can be drawn.

## 3. Discussion

Polyester polyol of the second generation, as the first representative of the homologous series, was chosen as the base polymer for this study [84]. In addition, the second generation polyol is the closest in structure to dendrimers, which is useful when discussing the results. As the Boltorn H series are polyesters in nature, this makes them biodegradable and biosimilar materials (Figure S1), as evidenced by their LD_50_~2000 mg/kg [85].

This is what allows them and their derivatives to be positioned for biomedical applications. The methods of chemical reduction (ChemRed), the polyol thermolytic process (TermRed), and the sonochemistry method (US) were selected as the most accessible and widespread for the synthesis of polymer-stabilized iron-based nanocomposites. The methods of chemical reduction, the thermolysis method, and the sonochemistry method were chosen as the most accessible and widely used methods for the synthesis of polymeric stabilized iron nanoparticles. Additionally, for the synthesis of a composite nanomaterial, a hybrid synthesis method was used (US/TermRed), which combines the simultaneous ultrasonic and thermal effects on the system for the formation of nanocomposites.

### 3.1. Synthesis of Iron-Based Composite Nanomaterials

The method of reduction in solution without the use of heating is convenient in technical and laboratory performance, as well as for the quantitative evaluation of products. The data [79] showed that the maximum sorption of metal ions by a branched polyester polyol is determined by the concentration of the terminal OH groups of the polymer. Therefore, a ratio of ν_OH_:ν_Fe2+_ = 1:1 was chosen for the synthesis. In a previous work, [79] showed that the maximum sorption of metal ions by a branched polyester polyol is determined by the concentration of the terminal OH groups of the polymer. Therefore, a ratio of 1:1 was chosen for the synthesis. 

By the ChemRed method, when the reducing agent is added color transitions of the solution are observed: the formation of a black, fine precipitate around a drop of hydrazine hydrate, which then turns into a green-blue suspension, changing color to lemon, and then to dark yellow. The end of the reaction was determined by the establishment of a stable, light cream color of the reaction mixture (Figure S2A). The total time for the synthesis of the nanocomposites by chemical reduction in a solution of ChemRed-NP powder was 30 min.

The color transitions observed during the synthesis indicate the formation of the following compounds in the system:

Fe^2+^/BH20 + N_2_H_4_ × H_2_O = Fe^0^/BH20 (black) + N_2_ + 2H_2_ + H_2_O (1)

Fe^0^ + 2H_2_O + O_2_ = 2Fe(OH)_2_/BH20 (green) (2)

Fe(OH)_2_/BH20 = FeO/BH20 + H_2_O (3)

4Fe(OH)_2_/BH20 + O_2_+ (2n − 4)H_2_O = 2Fe_2_O_3_ × H_2_O/BH20 (4)

The synthesis of TermRed-NP nanocomposites was carried out by reducing the precursor compound in a hyperbranched polyester polyol medium. To reduce the viscosity of the melt and increase the rate of the diffusion processes, DMSO was added as an additional solvent. The reduction process of Fe(II) HBP was carried out by heating the reaction system with a step of 5 °C. The heating step was 9 h, and the total synthesis time to stabilize the reaction mixture was 12 h. It can be assumed that, at the first stage of the reaction, the Fe(II) ions are preorganized in the polyol matrix with the formation of localized coordination sites. Ion binding can be carried out by the oxygen atoms of the peripheral ester and deprotonated hydroxyl groups of the polymer. With a further increase in temperature, a reduction stage occurs with the formation of polyol-stabilized nanoparticles. The nanocomposites obtained by the method of thermal reduction, TermRed-NP, in a solution of FeSO_4_ as a precursor, could not be isolated in the solid phase. The colors of the obtained material, TermRed-NP, are shown in the ESI (Figure S2B).

To avoid the use of toxic pentacarbonyl, a hybrid synthesis method based on iron sulfate 2 was proposed. Its essence lies in the most complete distribution of iron ions due to ultrasonic treatment, followed by reduction to nanoparticles by the polyol method. As the stage of the preorganization of the nanocomposites occurs under the action of ultrasound, the synthetic mixture was heated in one stage to 189 °C. The colors of the obtained material, US/TermRed-NP, are shown in the ESI (Figure S2C).

The ultrasonic method of obtaining a composite material (US-NP) does not apply to recovery. Ultrasonic treatment of the solution destroys the least stable component of the reaction mixture, in our case iron pentacarbonyl. After that, individual Fe0 atoms and clusters are stabilized by HBP in the form of nanoparticles. However, due to the small size and faster growth and stabilization processes, as well as the use of water as a solvent, the obtained nanocomposites do not have a uniform composition, which will be shown below. The colors of the obtained material, US-NP, are shown in the ESI (Figure S2D).

### 3.2. Characterization of Iron-Based Composite Nanomaterials by Microscopy

**Figure 1:**
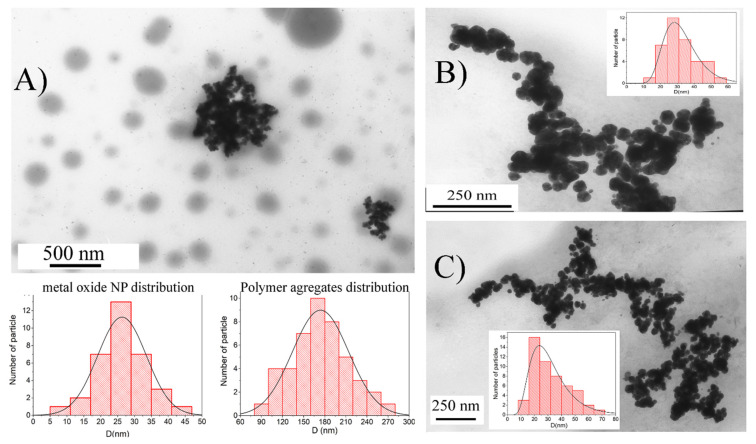
TEM image of particles ChemRed-NP (**A**), US/TermRed-NP (**B**), US-NP (**C**) with inserts of histogram of particle size distribution.

Using the TEM method, it was proved that the obtained samples of polymer composite materials contain a metal nanophase. Figure 1 shows the TEM images of the obtained particles in situ. For the TermRed-NP sample, it was not possible to obtain good TEM images due to the boiling of the sample during the measurement. For the TermRed-NP sample, it was not possible to obtain good TEM images due to the boiling of the sample during the measurement.

As was written in the synthesis section, the nanocomposites obtained by thermal reduction in the FeSO_4_ solution as a precursor could not be isolated in the solid phase. In addition, due to the synthesis in a high-boiling solvent, the sample could not be characterized by microscopic examination methods, since heating by an electron beam in a high microscope vacuum leads to the evaporation of the solvent together with the nanocomposites and the destruction of the sample holder (Lacey carbon).

Regardless of the production method, particles of a composite material of a spheroidal shape were formed. The average diameter of a metal-containing nanophase in the reaction medium was ~30 nm, and in the polymer fraction, it was −170 nm. The US/TermRed-NP sample (Figure 1B) had the narrowest particle size distribution. Regardless of the method of preparation, the particles of a composite material of a spheroidal shape were formed. The average diameter of the metal nanophase in the reaction medium was ~30 nm, and in the polymer fraction, it was −170 nm. The US/TermRed-NP sample (Figure 1B) had the narrowest particle size distribution.

**Figure 2:**
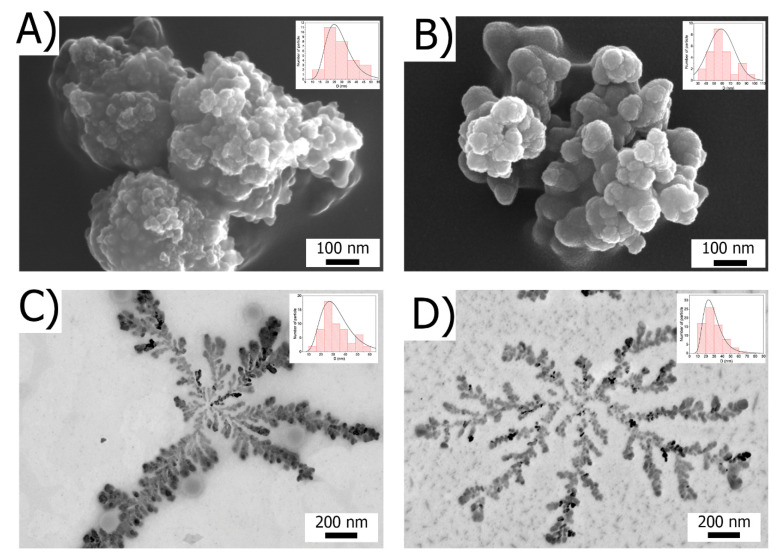
SEM images of particles ChemRed-NP (**A**), TermRed-NP (**B**), US/TermRed-NP (**C**), US-NP (**D**) with inserts of histogram of particle size distribution.

Scanning electron microscopy data were obtained for all the samples after purification (Figure 2).

The ChemRed-NP sample is represented by spherical aggregated particles with a diameter of 30 ± 5 nm, which correlates with the in situ TEM data. The TermRed-NP sample is similar in morphology but contains two fractions of particles with a diameter of up to 50 nm and 85 ± 10 nm. 

The US/TermRed-NP and US-NP samples self-assemble into dendritic structures on the substrate surface. The sizes of the individual particles forming a fractal are identical to those in the reaction mixture and are 25/55 nm and 25 nm for US/TermRed-NP and **U**S-NP, respectively. Confirmation of the statistical significance of the structures presented in Figure 2C,D are presented in the ESI section (Figures S3 and S4).

### 3.3. Stabilization and Composition of Iron-Based Nanomaterials

**Figure 3:**
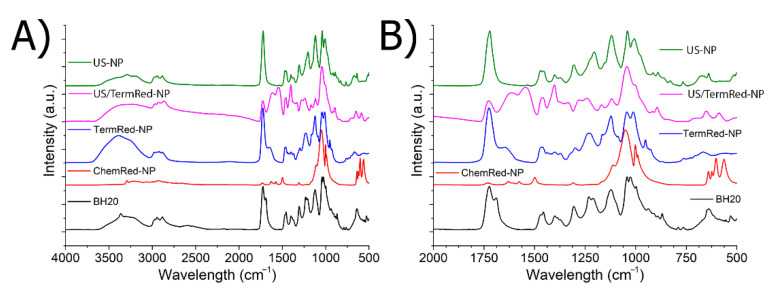
FT-IR spectra of hyperbranched polyester and samples ChemRed-NP, TermRed-NP, US/TermRed-NP, and US-NP: (**A**) full spectrum; (**B**) fingerprint area.

Establishing the nature of the nanocomposite stabilization centers in the structure of a branched polymer is one of the most important characteristics of a nanomaterial. The most convenient method for solving this problem is the IR-Fourier spectroscopy method [79]. Figure 3 shows the IR spectra of a branched polyester polyol BH20 and the iron-based composite nanomaterials based on it. 

The parameters of the IR spectrum of polyester polyol at room temperature and after heating were described in detail in the work of [86]. In the IR spectrum of the ChemRed-NP sample (Figure 3), strong changes were observed: the bands of the stretching and deformation vibrations of the bound and free OH groups were not pronounced, or were significantly decreased, in the region of 3560 cm^–1^ (ν_OH free_), 3450, 3350, 3250 cm^−1^ (ν_OH H-bonded_), and 1304 cm^−1^ (δ_OH H-bonded_); there were also stretching vibrations of the C=O ester groups at 1728 cm^−1^ (ν_C=O free_) and 1688 cm^−1^ (ν_C=O H-bonded_) and stretching vibrations of C-O and O-C ester groups at 1232 cm^−1^ (ν_ester C-O_) and 1166 cm^−1^ (ν_ester O-C_). It can be assumed that the stabilization of the particles of the metallic phase occurs due to the terminal OH groups and ester fragments -O-C(O)-. The appearance of a new band in the region of ~1630 cm^−1^ (Figure S5B) is due to the presence of water in the composition of the composite material, which can participate in the hydration of the C=O ester groups of [86]. The result of the partial hydration of the matrix polyester polyol is the formation of carboxyl groups. In the spectrum of the nanomaterial, there is a shift of these bands to the region of 1605 cm^−1^ (ν_COOH H-bonded_) and 1575 cm^−1^ (ν_COO-as_), which indicates their participation in the stabilization of the metal phase. 

In the IR spectrum of the TermRed-NP sample (Figure 3), the intensity of the bands of the stretching vibrations of the free and bound OH groups in the region of 3200–3600 cm^−1^ increased. The intensity of the band of free C=O groups at 1728 cm^−1^ and a shift H-linked C=O groups up to 1645 cm^−1^ was observed. These data indicate the participation of the peripheral OH groups and carbonyl groups of the ether fragments of the polymer in the stabilization of the iron nanophase.

For the US-NP sample, there was a significant decrease in the intensity of the bands of the stretching vibrations of the OH groups and the disappearance of the band of the stretching vibrations of the H-bonded C=O group of ester fragments (Figure S5D). It can be assumed that the centers of stabilization of the iron nanophase in the polymer are similar to those of the TermRed-NP sample. 

In the case of US/ThermRed-NP, there is a decrease in the intensity of the bands of the stretching vibrations of the OH-groups and a change and shift of the bands of the vibrations of the characteristic groups of the ester fragment in the region of 1728–1255 cm^−1^, including the hydration process. The data indicate the identity of the nanophase stabilization centers in the ChemRed-NP and US/TermRed-NP samples. 

**Figure 4:**
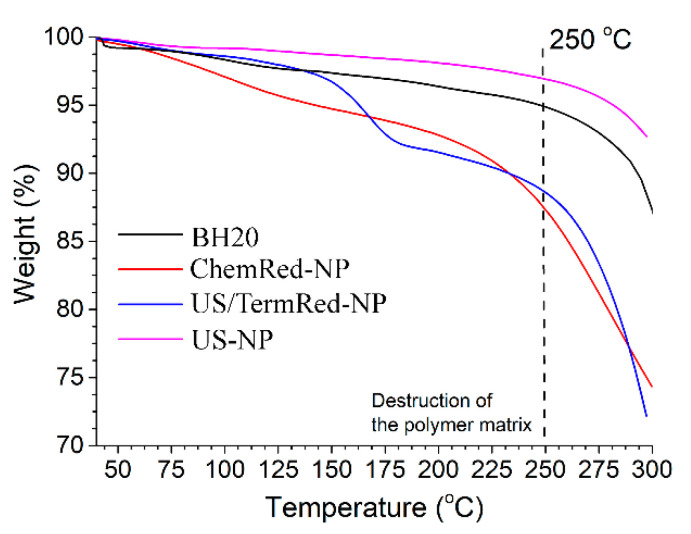
TG analysis of hyperbranched polyester BH20 and samples of ChemRed-NP, US/TermRed-NP, and US-NP.

The doping of the hyperbranched polyester polyol matrix with iron nanocomposites should lead to the effect of the doping of the composite material. The data of the TG analysis of the powdered samples of the nanocomposites (Figure 4) indicate that only the US-NP sample has the effect of doping with a metal phase. For the ChemRed-NP and US/TermRed-NP samples, the effects of mass change up to 5% in the range of 75–120 °C are due to the loss of the bound H_2_O (confirmed by IR data). An increase in temperature in the range of 140–190 °C leads to a significant weight loss, which is associated with the destruction of the polymer matrix.

**Figure 5:**
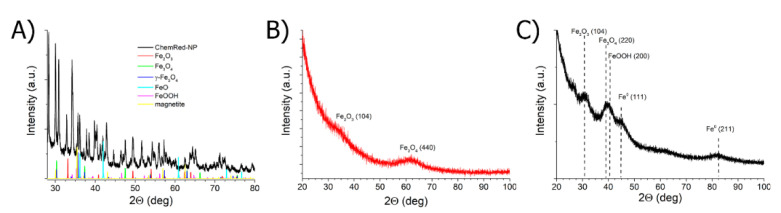
XRD spectra of (**A**) ChemRed-NP; (**B**) US/TermRed-NP; (**C**) US-NP.

XRD analysis was used to evaluate the composition of the iron nanophase in the synthesized samples of the composite material. The X-ray diffraction of polyol BH20 had one peak at 2θ = 17.6 (Figures S6 and S7). The authors showed that the polymer stabilizer has a close packing of chains; the distance between the chains is 0.509 nm. The number and thermal stability of the -OH...-OH hydrogen bonds are higher than those of the -OH...O-C < H-bonds. The TermRed-NP sample was not isolated as a powder since it has a high colloidal stability. This state may be due to the high content of the polymer phase. Therefore, XRD analysis was performed only for the three samples of ChemRed-NP, US/TermRed-NP, and US-NP. As can be seen from the XRD spectrum (Figure 5A), the metal nanophase in ChemRed-NP contains almost all the types of iron oxides. 

In the case of US-NP (Figure 5C), the diffraction pattern contains the α-Fe^0^ reflection at angles of 2θ = 45 and 82, as well as signals from the oxide phase. It can be assumed that the metal phase nanocomposites have a core (Fe^0^)–shell (iron oxides) structure. Upon passing to the nanomaterial obtained by the US/TermRed-NP hybrid method (Figure 5B and Figure S8), a significant decrease in crystallinity is observed. On the diffraction pattern, only the broadened signal of magnetite can be distinguished at angles of 2θ = 62.

**Figure 6:**
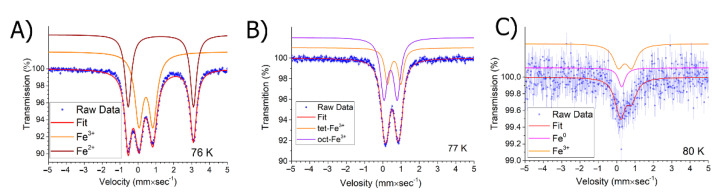
Mossbauer spectra of (**A**) ChemRed-NP; (**B**) US/TermRed-NP; (**C**) US-NP.

Our conclusions on the nature of the metallic phase are confirmed by Mössbauer spectroscopy data (Figure 6).

The spectra obtained at room temperature are presented in the ESI section (Figures S9–S11). As can be seen in Figure 6C, the core-shell metal phase is present only in the US-NP sample, which confirms the XRD data. The shape of the spectrum indicates small particle sizes. The spectra of the ChemRed-NP and US/TermRed-NP nanocomposites contain only the signals of the oxidized forms. 

### 3.4. Magnetic Properties of Iron-Based Composite Nanomaterials in Solid State and Solution

**Figure 7:**
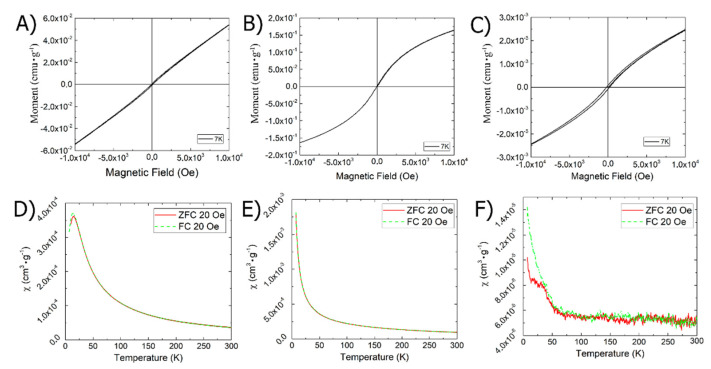
Magnetic hysteresis: (**A**) ChemRed-NP, (**B**) US/TermRed-NP, (**C**) US-NP; FC/ZFC curve: (**D**) ChemRed-NP; (**E**) US/TermRed-NP; (**F**) US-NP.

MRI is currently the most convenient, common, and complete diagnostic method. As contrast agents are primarily supplied through the blood, they must have two key characteristics: they must be magnetically susceptible and non-toxic, primarily to the blood. First of all, we checked the magnetic susceptibility of the obtained materials; Figure 7 shows the dependencies of the magnetization of the samples on the magnitude of the external magnetic field (A, B, and C) and the dependencies of the magnetic susceptibility on temperature at an external magnetic field of 20 Oe (D, E, F) in the obtained materials.

As can be seen in Figure 7, the ChemRed-NP, US/TermRed-NP, and US-NP samples at a temperature of 7 K have a coercive force of 100, 30, and 200 Oe, a remanent magnetization of 0.03, 0.05, and 0.0038 emu × g^−1^, and saturation magnetization hysteresis loops of 2.58, 5.77, and 0.107 emu × g^−1^, respectively.

The temperature dependence of the susceptibility in the FC mode of the samples increases with the decreasing temperature in accordance with the Curie–Weiss law, with critical temperatures of −13, −3, and −6 K, which indicates a ferrimagnetic phase.

**Figure 8:**
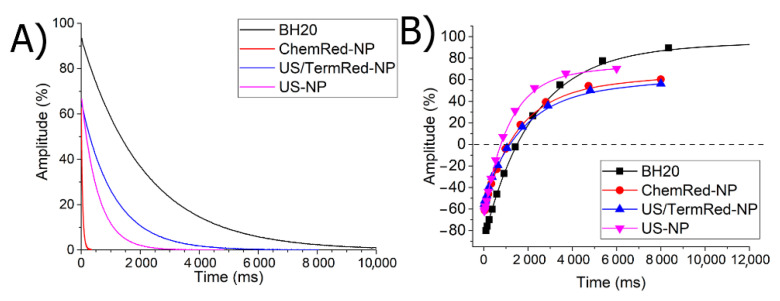
(**A**) Transverse dependence of relaxation on time; (**B**) longitudinal dependence of relaxation on time.

The translation and preservation of the material properties from the solid phase to the solution is one of the most difficult tasks of the science of materials. If we want to use the resulting magnetic nanocomposites as an MRI contrast agent, then they must retain their magnetic characteristics in solution. To do this, all the obtained nanocomposites were repeatedly transferred to the solution; water-retaining DMSO was used as a solvent. Figure 8 shows the curves of magnetic relaxation in a solution of the obtained nanocomposites.

The data obtained confirm that ChemRed-NP and US-NP retain their magnetic properties after solidification and are classified as ferrimagnets (Figure 8), which would allow them to be used as contrast agents for magnetic tomography.

### 3.5. Aggregation Properties of Iron-Based Composite Nanomaterials

**Figure 9:**
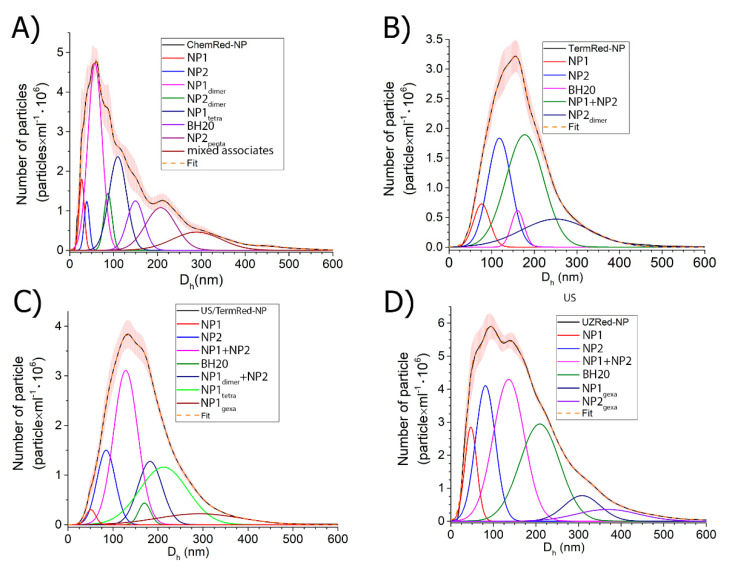
NTA analysis of nanocomposites dispersion: (**A**) ChemRed-NP (RMSE = 0.9995; χ^2^ = 7.6 × 10^−4^); (**B**) TermRed-NP (RMSE = 0.9998; χ^2^ = 1.5 × 10^−4^); (**C**) US/TermRed-NP (RMSE = 0.9999; χ^2^ = 3.3 × 10^−5^); (**D**) US-NP (RMSE = 0.9993; χ^2^ = 6.5 × 10^−4^) in aqueous solutions (cNP = 0.1 mg·mL^−1^).

The further use of iron-based nanocomposites in biomedicine requires information on the morphology of the particles of the dispersed phase in solution. The NTA method was used to evaluate the behavior of the synthesized nanocomposites in an aqueous solution. (Figure 9). 

The NTA method showed that in a BH20 solution with a concentration of 0.1 mg·mL^−1^ there were two types of closely related associates, with a hydrodynamic diameter of 150 ± 8 nm. As can be seen in Figure 9, when redispersed all the colloidal solutions contain a fraction of the free polymer aggregates, which are formed during the desorption of the weakly bound polymer molecules from the surface of the nanocomposite. The concentration (in particles × mL^−1^) of free BH20 aggregates increases in the series ChemRed-NP (1.8 × 10^6^) < TermRed-NP (3.2 × 10^6^)~US/TermRed-NP (3.0 × 10^6^) < US-NP (4.4 × 10^6^) (Figures S12–S15). The average hydrodynamic diameter of the ChemRed-NP particle aggregates is slightly smaller compared to the particle aggregates obtained by the other methods. Detailing the NTA data showed that the aqueous dispersions of all the synthesized composites contain two types of metal–polymer aggregates, with the hydrodynamic diameters D_h_(mode) of 27 and 40, 77 and 118, 50 and 80, and 35 and 52 nm for the ChemRed-NP, TermRed-NP, US/TermRed-NP, and US-NP samples, respectively (Figure 9).

Thus, the distribution of the nanocomposites in the solid phase obtained by TEM and SEM correlates with the distribution of the nanocomposites in solution (Table 1).

The data obtained are useful for the development of the nanocomposite solubilization techniques.

### 3.6. Hemolytic Activity Assay

**Figure 10:**
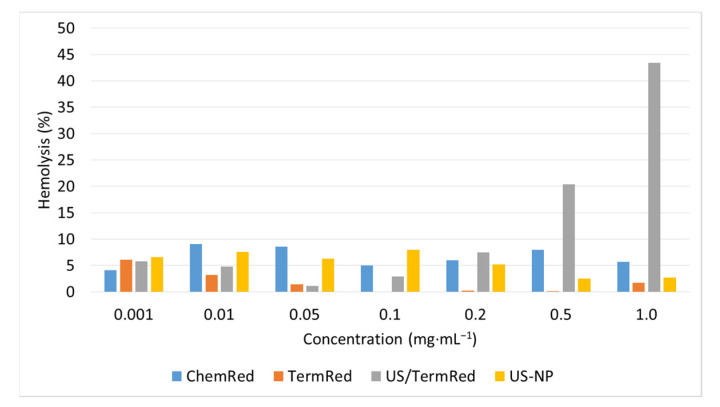
Hemolysis of the ChemRed-NP, TermRed-NP, US/TermRed-NP, and US-NP.

The main route of administration of contrast agents is intravenous. Because of this, the blood is exposed to the strongest toxicological effects. In order to confirm the applicability of the obtained composite materials for biomedical use, their hepatotoxicity was evaluated (Figure 10).

It was found that in the concentration range of 0.001–1 mg·mL^−1^, the composite ChemRed-NP, TermRed-NP, and US-NP nanocomposites had low hemolytic activity. The minimum average value of hemolytic activity of 2.2 ± 1.9% was observed for the TermRed-NP sample, with a particle size in the iron nanophase of 50 nm. The ability to cause hemolysis slightly increases with a rise in the diameter of the nanocomposites to 25 nm, and it was 6.6 ± 1.8% and 5.5 ± 2.2% for the samples of ChemRed-NP and US-NP, respectively. The US/TermRed-NP is not hemotoxic in the concentration range of 0.001–0.2 mg·mL^−1^. A rise in concentration leads to an increase in hemotoxicity of up to 45%.

## 4. Materials and Methods

### 4.1. Materials

As precursors for the synthesis of the nanoparticles of the iron compounds, we used FeSO_4_·7H_2_O (Alfa Aesar GmbH. Kandel, Germany), Fe(CO)_5_ (mass fraction of the main substance 99.99%, Sigma Aldrich, Darmstadt, Germany), and Boltorn hyperbranched polyester polyols H series (Perstorp Specialty Chemicals AB, Perstorp, Sweden): H20 (CAS no: 326794-48-3, lot. 369087, 16 hydroxyl groups, M_r_ (theor) = 1749 g·mol^−1^, hydroxyl number 490–520 mg KOH × g^−1^) (Figure S1). As solvents for the synthesis and isolation of iron-containing nanocomposites, organic solvents were used: ethanol, DMSO, methanol, diethyl ether, and acetone, purified according to the standard procedures.

### 4.2. Synthesis Methods

#### 4.2.1. Chemical Reduction (ChemRed-NP)

Boltorn H20 (ν_OH_ = 6.13 × 10^−3^ mol) was dissolved by stirring in 6 mL of an aqueous ethanol solution at room temperature. Then, a solution of 1.73 g (ν_Fe2+_ = 6.22 × 10^−3^ mol) FeSO_4_·7H_2_O was added dropwise in 6 mL of a water–ethanol solution; the salt–polymer mixture was stirred for 20 min. Then, 1 mL of N_2_H_4_·H_2_O was added until a stable light brown color appeared, and the reaction mixture was centrifuged (v = 14,000 rpm, t = 10 min). The resulting powder composition containing iron nanocomposites was freed from the solvents by reprecipitation in diethyl ether. The supernatant, also containing polymer-stabilized nanocomposites of iron compounds in solution, was an independent sample and was used for the study (Figure S2A).

#### 4.2.2. Polyol Thermolytic Process (TermRed-NP)

The Boltorn H20/FeSO_4_·7H_2_O ratio was ν_OH_ = 6.13 × 10^−3^ mol/ν_Fe2+_ = 6.05 × 10^−3^ mol. The samples were dissolved in 25 mL of preliminarily purified DMSO; then, the reaction mixture was boiled at 189 °C for 12 h. To the cooled mixture was added a 25 mL solution of H_2_O/0.1 M HCl (1:1) to prevent hydrolysis. The resulting brown-yellow slurry was centrifuged (Figure S2B). After the precipitate was redispersed in ethanol (98%) and centrifuged, the procedure was repeated three times. Despite the procedures performed, it was not possible to remove the DMSO from the obtained material.

#### 4.2.3. Hybrid Ultrasonic/Thermolytic (US/TermRed-NP)

The Boltorn H20/FeSO_4_·7H_2_O ratio was ν_OH_ = 6.13 × 10^−3^ mol/ν_Fe2+_ = 6.05 × 10^−3^ mol; it was dissolved in preliminarily purified DMSO, and 6.05 × 10^−3^ mol Fe(CO)_5_ was added into the solution by stirring. The resulting mixture was sonicated for two hours in pulse mode 2/2 with stirring. After ultrasonic treatment, the mixture was boiled for 4 h at 189 °C and was added to a 25 mL solution of H_2_O/0.1 M HCl (1:1). The light yellow suspension was formed (Figure S2C) and then centrifuged. The precipitate was redispersed in ethanol (98%) and centrifuged; the procedure was repeated three times.

### 4.3. Ultrasonic (US-NP)

A weighed portion of BH20 (ν_OH_ = 6.13 × 10^−3^ mol) was dissolved in a 50% ethanol/water solution by stirring and subjected to ultrasonic treatment in an argon atmosphere, on an ultrasonic bath. Then, Fe(CO)_5_ (ν_Fe0_ = 6.05 × 10^−3^ mol) was added dropwise to the system and sonicated until a stable color was established. After adding the FeCO_5_ solution, 95.5 kJ of energy was transferred for 6 h, in a 2/2 pulse mode, by a probe ultrasonic homogenizer, by Sonics VCX-750. The resulting mixture was centrifuged, and a yellow precipitate was obtained (Figure S2D). Then, the precipitate was redispersed in ethanol (98%) and centrifuged. This procedure was repeated 3 times.

### 4.4. Characterization Methods

The scanning electron microscopy (SEM) images were acquired with the field-emission high-resolution scanning electron microscope Merlin from Carl Zeiss (Jena, Germany) at an accelerating voltage of incident electrons of 5 kV and a current probe of 300 pA. The transmission electron microscope (TEM) imaging was carried out in the transmission electron microscope Hitachi HT7700 (Tokyo, Japan) Excellence at an accelerating voltage of 100 kV in the TEM mode.

The powder X-ray diffraction (XRD) was acquired with the Bruker D8 Advance (Bruker Corp., Billerica, MA, USA) with Cu Kα irradiation (λ = 1.5418 Å) in the Bragg–Brentano geometry; the rate was 0.18°/min; the range of the 2θ angle was from 7° to 100°; the step was 0.015°.

Simultaneous TG analyses were performed using a thermal analyzer STA 449 C Jupiter (Netzsch, Germany) with the temperature rate of 10 K × min^−1^ in an argon atmosphere with the total flow rate of 20 mL·min^−1^. The analysis was performed in a temperature range of 30–900 °C in a Pd/Rh crucible with a volume of 40 µL with a lid with 1 hole with a diameter of 0.5 mm at constant heating rates (10 K × min^−1^ and 4 K × min^−1^).

The Mössbauer effect measurements were carried out at room temperature, using a conventional constant-acceleration spectrometer, produced by WissEl (Germany). A commercial Mössbauer source of ^57^Co in a rhodium matrix (Ritverc isotope products, Saint Petersburg, Russia), with an activity of about 40 mCi, was used as the *γ* -radiation source. Low-temperature measurements were carried out with a continuous flow cryostat (model CFICEV from ICE Oxford, Oxford, UK), equipped with Cryo-Con temperature controller (Model 32B); the sample temperature was kept with an accuracy of ±0.1 K. The absorber was prepared by uniformly packing the sample under study into a holder closed by thin aluminum foil. The experimental spectra were least-squares fitted with the assumption that the line shapes were Lorentzian to yield the hyperfine parameters, namely the isomer shift (IS), quadrupole splitting (QS), and hyperfine field (H_HF_). A metallic-iron foil at RT was used for the velocity calibration of the Mössbauer spectrometer. The isomer shifts were referred to *α*-Fe at RT.

The colloidal properties were studied by the Nanoparticle Tracking Analysis (NTA) method on a NanoSight LM − 10 instrument (Malvern Panalytical, Malvern, England). The CMOS camera C11440-50B with an image capture sensor FL-280 from Hamamatsu Photonics (Hamamatsu, Japan) was used as a detector. The measurements were carried out in a special cuvette for aqueous solutions, equipped with a laser with a wavelength of 405 nm (CD version S/N 2990491); the O-ring was made of Kalrez material. The temperature in the chamber was determined using a contact thermometer OMEGA HH804 (Engineering Inc., Norwalk, CT, USA) for all measurements. For the spectra fitting, the OriginPro program package was use; the Gauss function was used throughout.

The magnetic properties of the iron nanocomposites were measured using a PPMS-9 (Quantum Design, San Diego, CA, USA) equipped with a sample vibration magnetometer (VSM). Zero-field-cooling (ZFC) and field-cooling (FC) measurements were performed in a 20 Oe magnetic field at the temperature range of 7–300 K. The field dependences of the magnetization were measured at 7 K in the magnetic field range from −1 T to 1 T.

The proton spin-spin (transverse) relaxation times T_2_ were measured using the pulsed NMRrelaxometer Minispec MQ20 from Bruker (Bruker Corp., Billerica, MA, USA), with the operational frequency of 19.65 MHz, by applying the standard Carr–Purcell pulse sequence modified by Meiboom and Gill with a measuring accuracy error smaller than 3%. The experimentally measured relaxation times, (T_2_)obs, were inverted into the relaxation rates, (1/T_2_)obs. The relaxation rate is the sum of the two main contributions: the relaxation of protons in the bulk solvent (1/T_2_)d (diamagnetic component) and the relaxation of the protons located in the first coordination sphere of the paramagnetic ion (1/T_2_)p (paramagnetic component): (1/T_2_)obs = (1/T_2_)d + (1/T_2_) [87].

### 4.5. Hemolysis Assay

The hemolysis assay was performed according to the method in [88]. Blood from healthy donors was obtained from the blood donor center in Kazan and was anticoagulated with 3% sodium citrate. The erythrocytes were separated from the plasma and leukocytes by centrifugation (5000× *g*, 5 min) at 4 °C and washed three times with phosphate-buffered saline (PBS). They were used immediately after isolation. To study the effects of the nanocomposites on hemolysis, the red blood cells (RBC) were suspended in polymer solutions in PBS at a hematocrit of 1% and incubated for 0.5 h at a temperature of 20 °C. The incubated suspensions were centrifuged at 1000× *g* for 5 min. For reference, the RBC were treated with double-distilled water, which effects 100% hemolysis. The hemolysis (%) was determined from the released hemoglobin in the supernatants and measured spectrophotometrically by absorbance at 540 nm [89]:

$$\mathrm{Hemolysis}\;\left[\%\right]={\frac{A-A_{-}}{A_{+}+A}}_{-}\times 100\%$$

where A is the optical density of the RBC incubated with nanocomposites, A− is the optical density of the sample in PBS, and A+ is the optical density of the RBC in water (100% hemolysis). The nanocomposites themselves contributed no more than 0.1% of the absorbance at 540 nm. The results are expressed as mean ± standard deviation, n = 5.

## 5. Conclusions

The paper considers four approaches (the chemical reduction method—ChemRed, the polyol thermolytic process—TermRed, the sonochemistry method—US, and the hybrid sonochemistry/polyol process method—US/TermRed) to the formation of an iron nanophase in a matrix of hyperbranched polyester polyols. The dendrit-like branched polyester polyol exhibits the properties of a reliable stabilizer of iron nanocomposites, and in the polyol process and the hybrid method, it also acts as a reducing agent. The stabilization of the iron nanophase occurs due to the interaction with the ester -O-C(O)- of the inner hydrophobic core and the OH groups of the hydrophilic shell of the polyol in the ChemRed and hybrid US/TermRed methods. The application of the TermRed and US methods gives stabilization not on the whole ester fragment, but only through its carbonyl C=O group at the periphery of the macromolecule. This leads to the thermal doping of the nanomaterial obtained by sonochemistry. The size and composition of the iron nanophase in the polyester polyol matrix can be controlled by choosing the synthesis method. Iron oxide particles with a diameter of 25 nm can be obtained in the ChemRed and US/TermRed methods. Carrying out high-temperature synthesis by the TermRed and US/TermRed methods leads to an increase in the diameter of the iron-based nanophase up to 50 nm. The use of sonification makes it possible to achieve the composition of the nanophase core (Fe^0^)–shell (iron oxides) with better dispersion characteristics and leads to the formation of dendritic composite architectures on the surface.

The authors suggest that the magnetic properties of the synthesized metal–polymer composites are determined primarily by the nature and mass fraction of the iron nanophase. So, in TermRed-NP the proportion of polymer was high, which did not allow them to be isolated from the colloid solution. The XRD profile for US/TermRed-NP may be indicative of an amorphous sample containing minor amounts of low-sized iron clusters. Therefore, the magnetic activity of this sample in the powder and the colloidal solution is low. According to the XRD data, the ChemRed-NP and US-NP samples have a sufficiently crystallized iron nanophase. This can lead to an increase in the residual magnetization by an order of magnitude. In addition, US-NPs containing Fe^0^ in the metal nanophase (in the core) have a higher magnetic activity than ChemRed-NPs, in which the metal nanophase is only magnetite.

All the synthesized composite particles are able to form colloidally stable systems, are ferrimagnets, and retain their magnetic properties after repeated dissolution. The most technologically and practically significant methods, in our opinion, are the methods of the chemical and sonochemical synthesis of composite magnetically active materials containing iron nanoparticles and hyperbranched polyester polyols. The synthesis of magnetoactive materials using the polyol process (TermRed and US/TermRed) has significant prospects. It has been shown for the first time that hyperbranched polyester polyols can act both as a reducing agent and as a stabilizer in the one-pot synthesis of nanomaterials. Experimental work in this project will continue.

The conducted experiments on hematotoxicity and relaxation NMR show the fundamental possibility of using a magnetically active composite material obtained by ultrasonic synthesis as the basis of contrast agents for tomography.

## Figures and Tables

**Table 1:** Dependence of the size (Dh) of a particle of iron-based composite nanomaterials obtained by various methods, according to NTA, SEM data, and their concentration in a solution.

Sample	D_h_(mean) by NTA; (nm)	D_h_(mode) by NTA; (nm)	D by SEM; (nm)	Total Particle Concentration in Solution; (Particles × mL^−1^)
ChemRed-NP	133 ± 7	27, 40	25	6.24 × 10^8^
TermRed-NP	172 ± 2	77, 118	50, 85	4.92 × 10^8^
US-NP	169 ± 6	50, 80	30, 55	1.25 × 10^9^
US/TermRed-NP	168 ± 4	35, 52	25	6.03 × 10^8^

## Supplementary Materials

The following supporting information can be downloaded at: https://www.mdpi.com/article/10.3390/ijms232314764/s1.
