# Supplementary material for: Effect of the Synthetic Approach on the Formation and Magnetic Properties of Iron-Based Nanophase in Branched Polyester Polyol Matrix
Source: Int J Mol Sci. 2022 Nov 25;23(23):14764. doi: 10.3390/ijms232314764 (PMC9735957; doi:10.3390/ijms232314764)
Supplement: Supplementary file 1 [file ijms-23-14764-s001.zip › ijms-2028565-supplementary.pdf]

Effect of the Synthetic Approach on the Formation and Magnetic Properties of Iron-Based Nanophase in Branched Polyester Polyol Matrix

Artur Khannanov [1], Anastasia Burmatova [1], Klara Ignatyeva [1], Farit Vagizov [2], Airat Kiiamov [2,*], Dmitrii Tayurskii [2], Mikhail Cherosov [2], Alexander Gerasimov [1], Evtugyn Vladimir [3] and Marianna Kutyreva [1]

[1] Butlerov Chemistry Institute, Kazan Federal University, Kazan 420008, Russia
[2] Institute of Physics, Kazan Federal University, Kazan 420008, Russia
[3] Interdisciplinary Center "Analytical Microscopy", Kazan Federal University, Kazan 420008, Russia
[*] Correspondence: ajrat.kiyamov@kpfu.ru

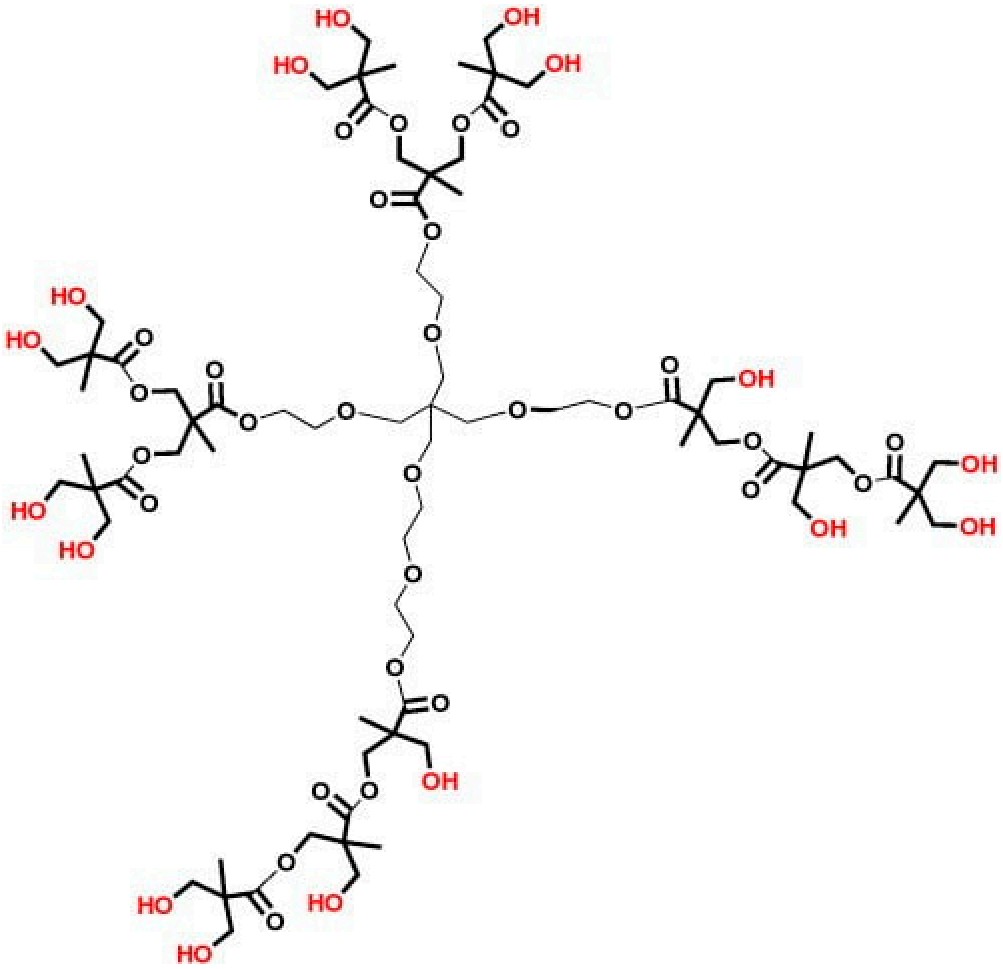

Figure S1. Theoretical structure of Boltorn H20

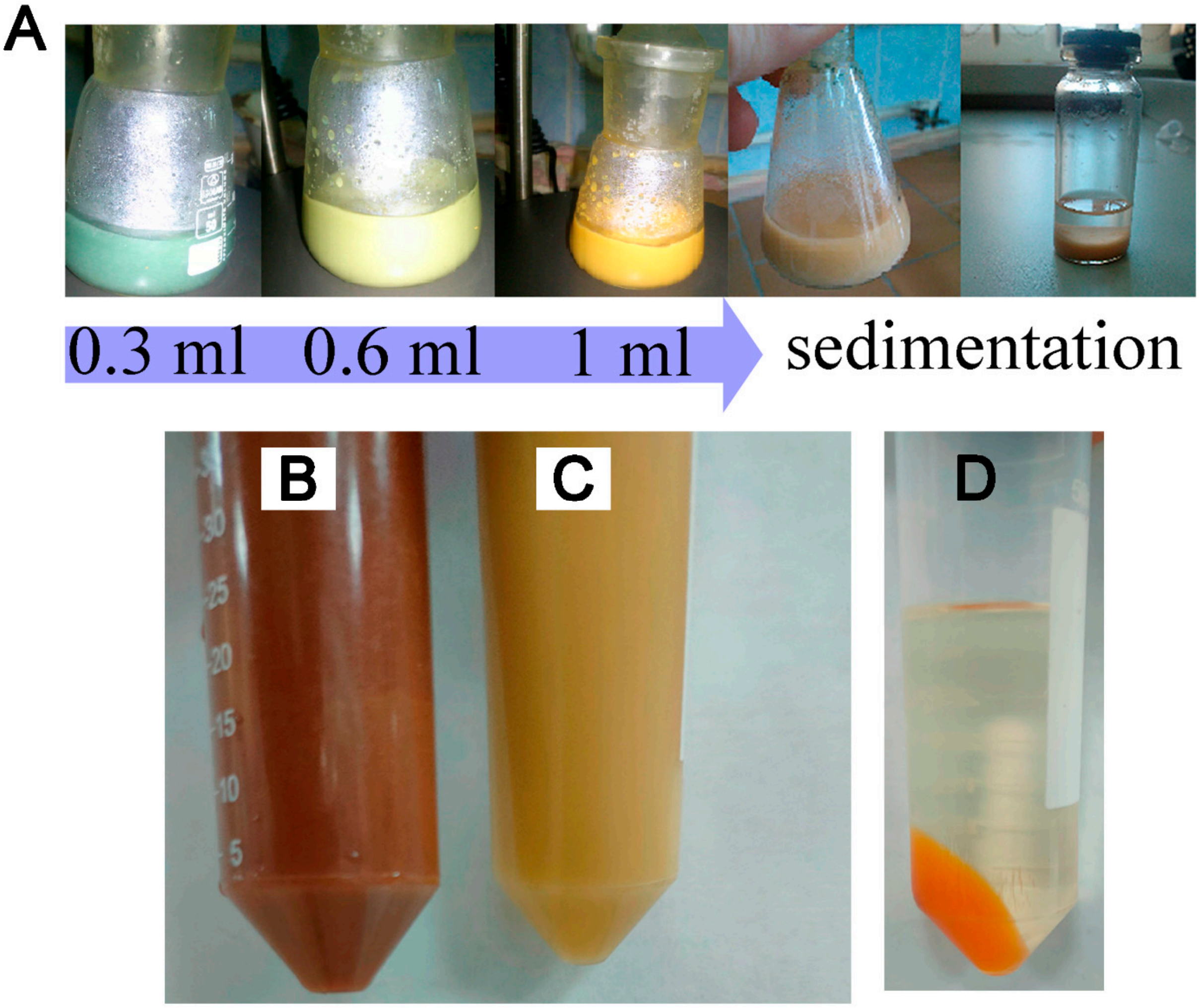

Figure S2. A) ChemRed-NP Change in the color of solutions during the synthesis of Fe_mO_n/H20 nanoparticles with the addition of $N_2H_4{\times}H_2O$ and color of the dispersed system containing composite Fe_mO_n/H20 nanoparticles at the end of the synthesis reaction; B) TermRed-NP reaction mixture during washing before centrifugation; C) US/TermRed-NP reaction mixture during washing before centrifugation;; D) US-NP after centrifugation.

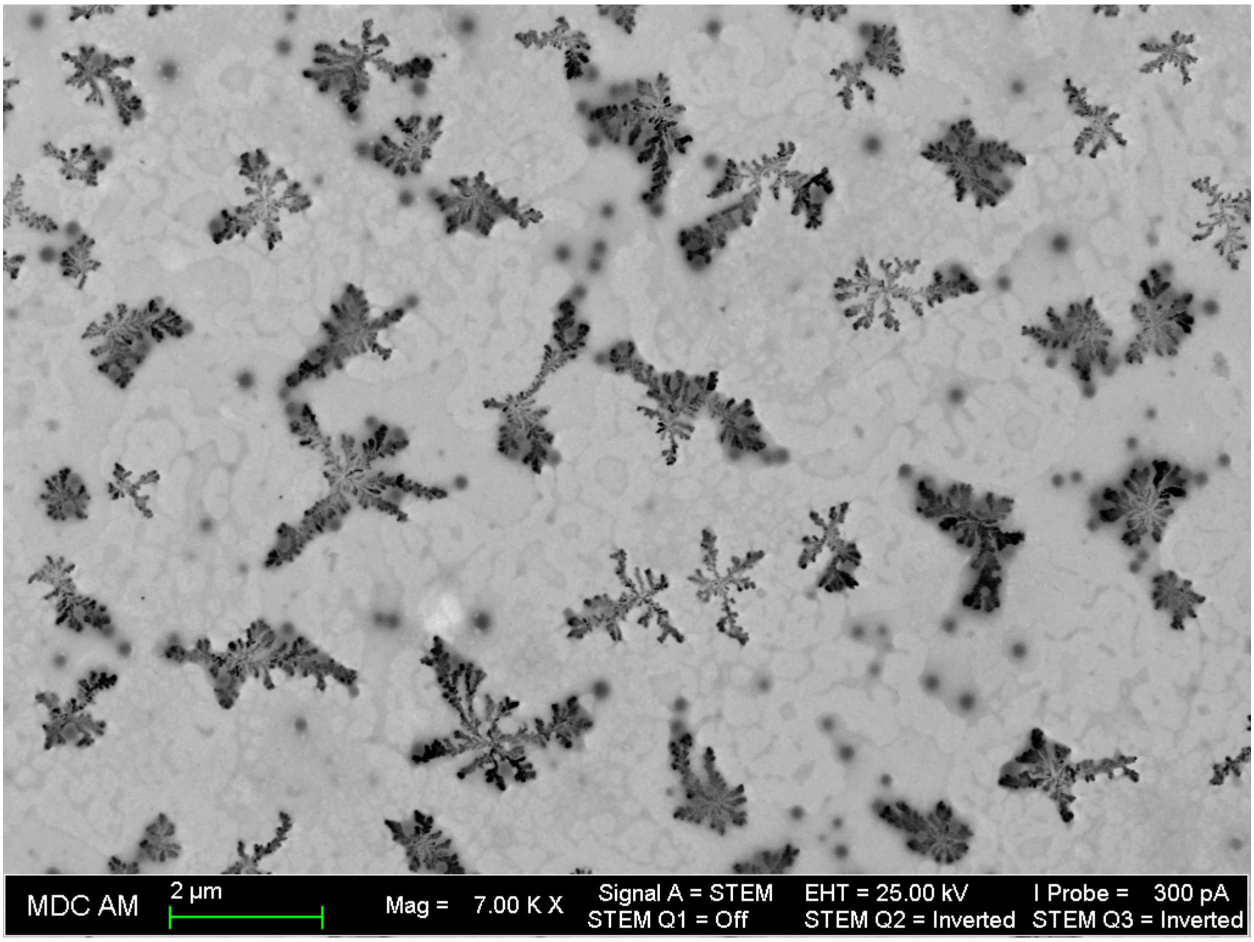

FigureS3. SEM image of US/TermRed-NP nanoparticles to confirm the statistics.

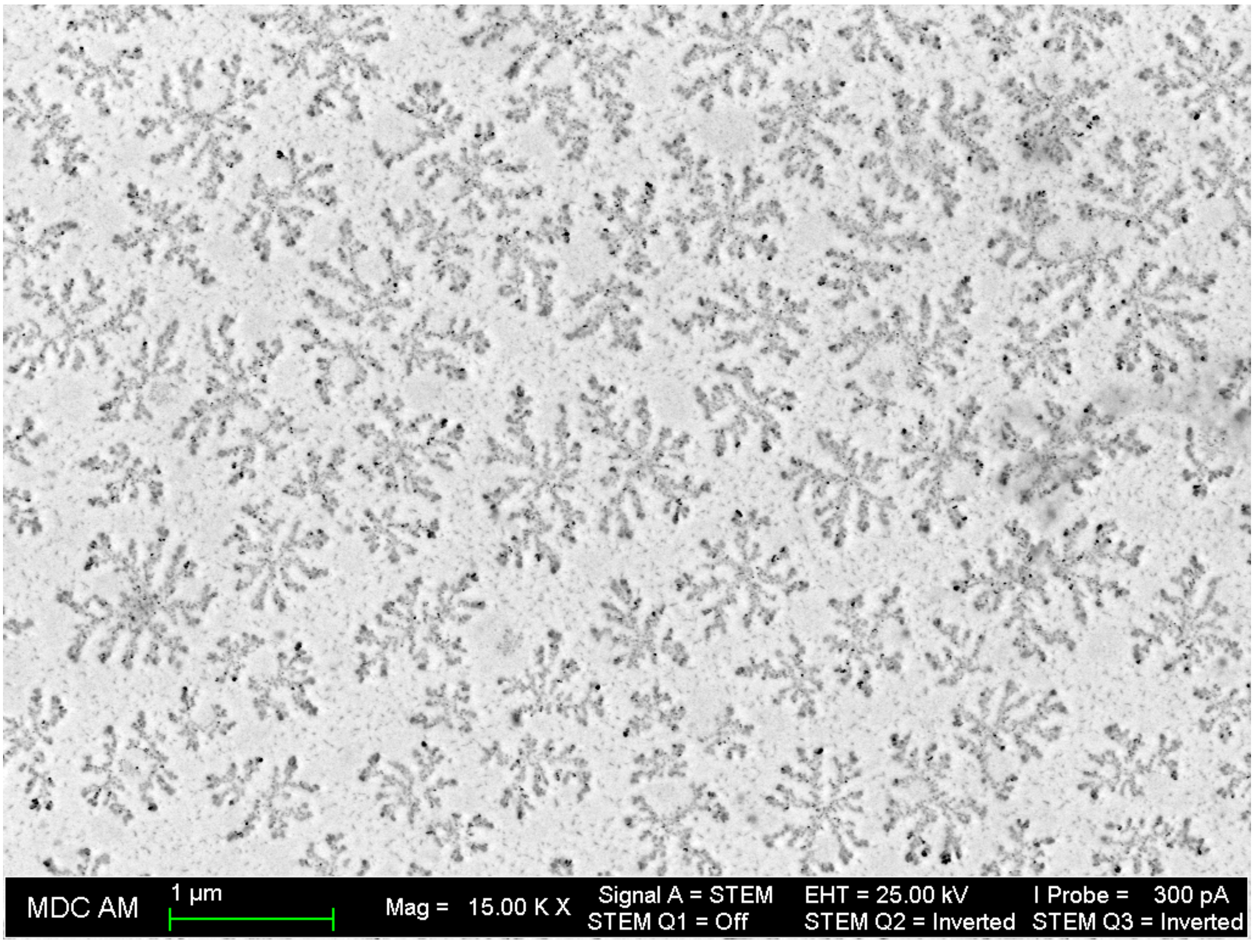

FigureS4. SEM image of US-NP nanoparticles to confirm the statistics.

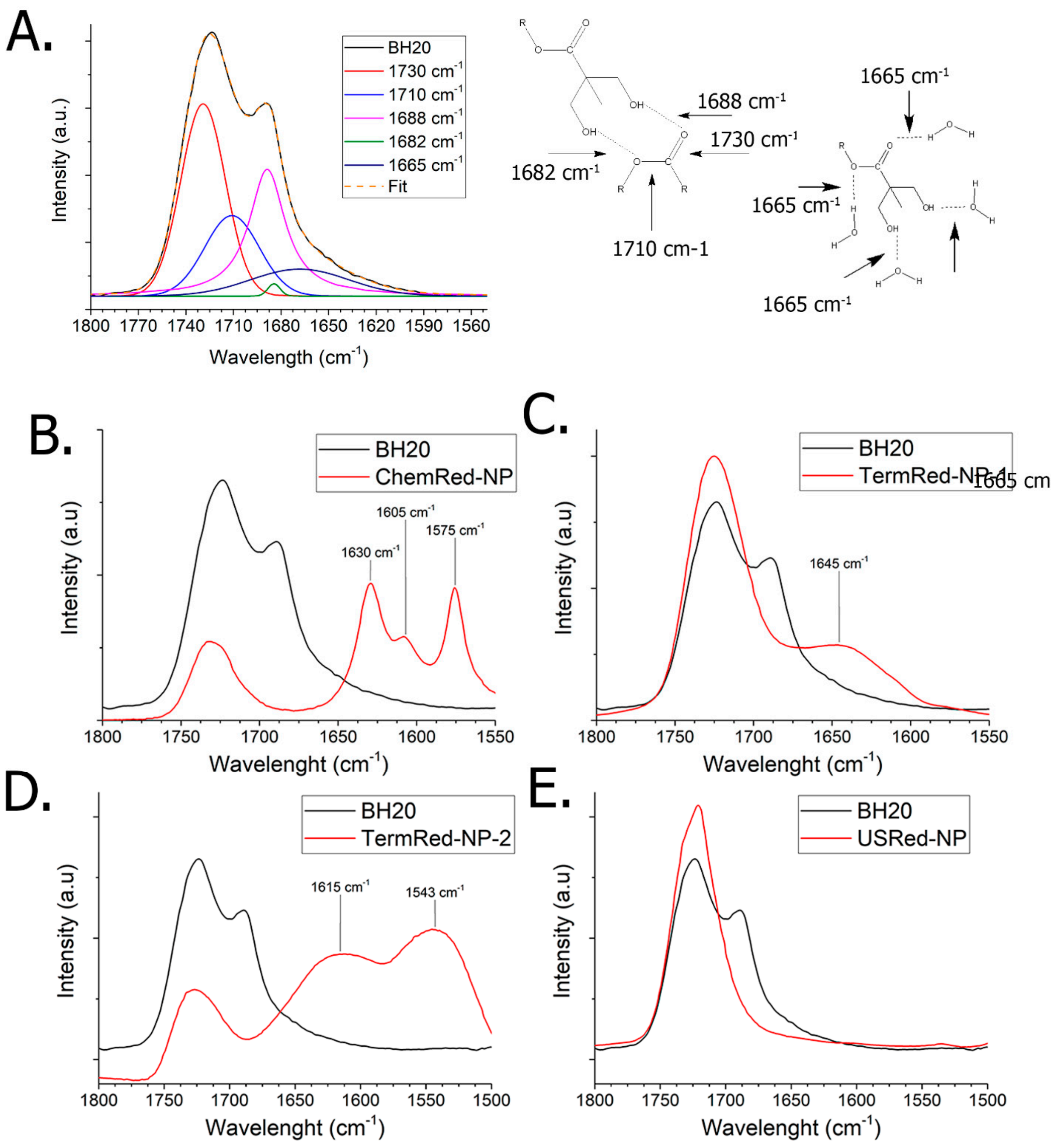

FigureS5. Ft-IR spectra: A) BH20; B) ChemRed-NP; C) TermRed-NP; D) US/TermRed-NP; E) US-NP.

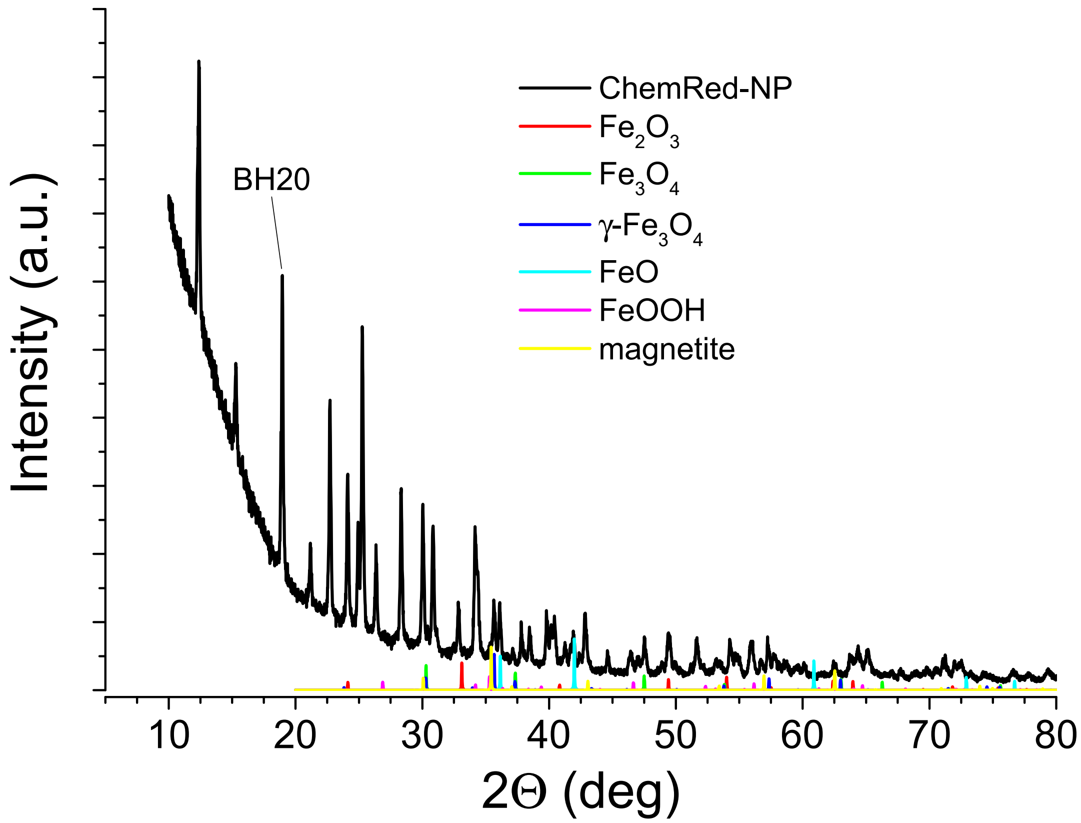

FigureS6 Full XRD spectra of **ChemRed-NP**.

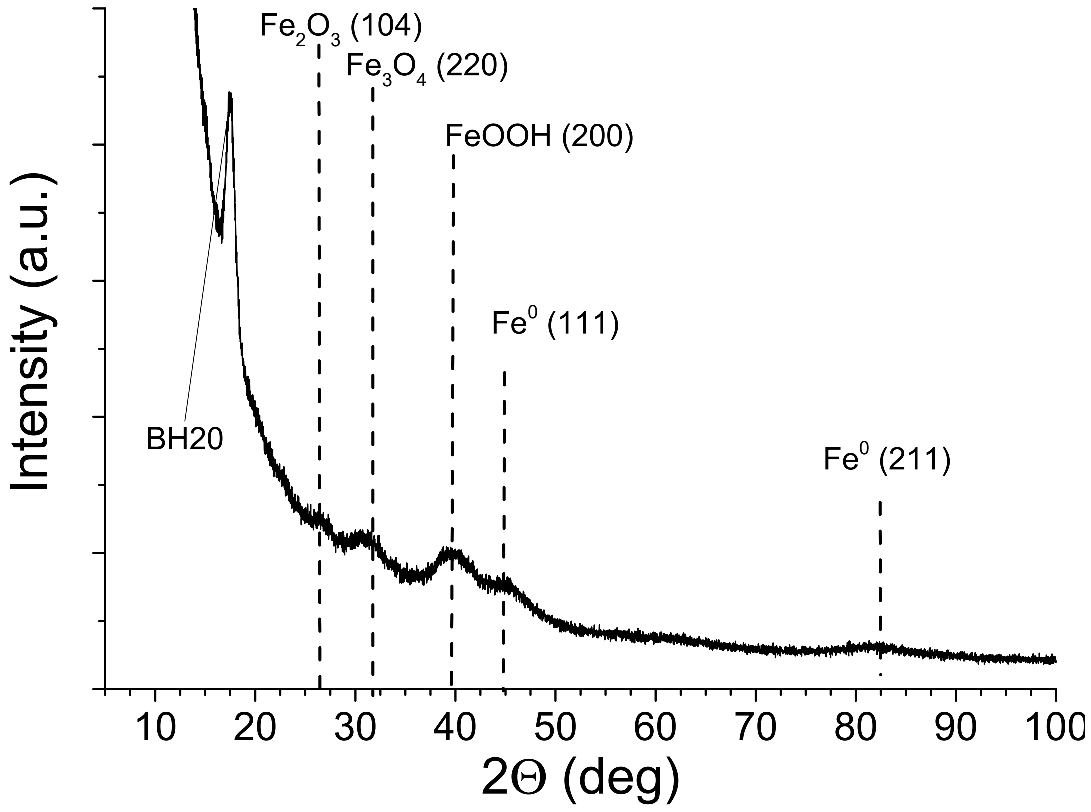

FigureS7 Full XRD spectra of **US-NP**.

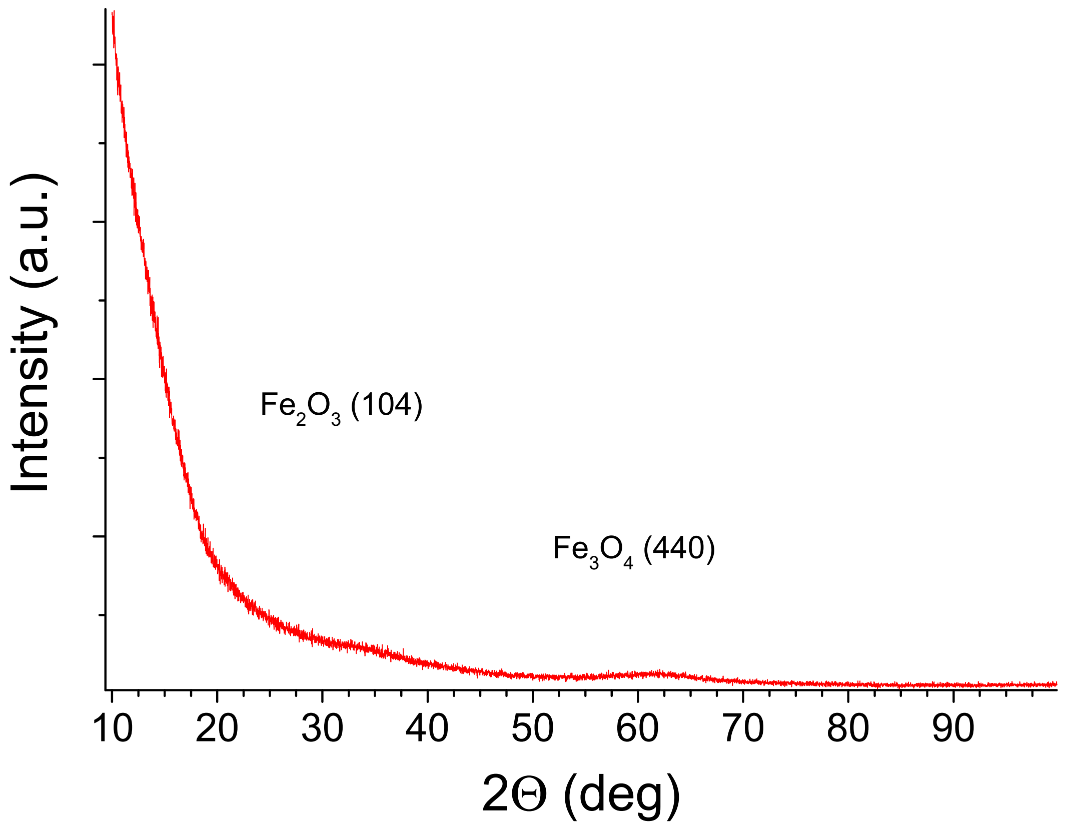

FigureS8. Full XRD spectra of **US/TermRed**.

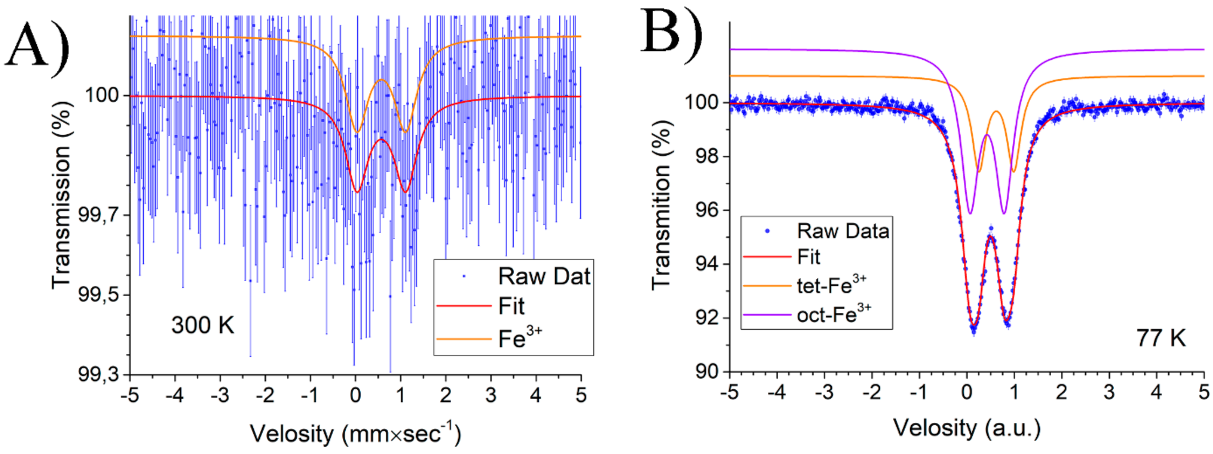

Figure S9 Mossbauer spectra of US/TermRed-NP with deconvolution: A) at 300 K; B) at 77 K.

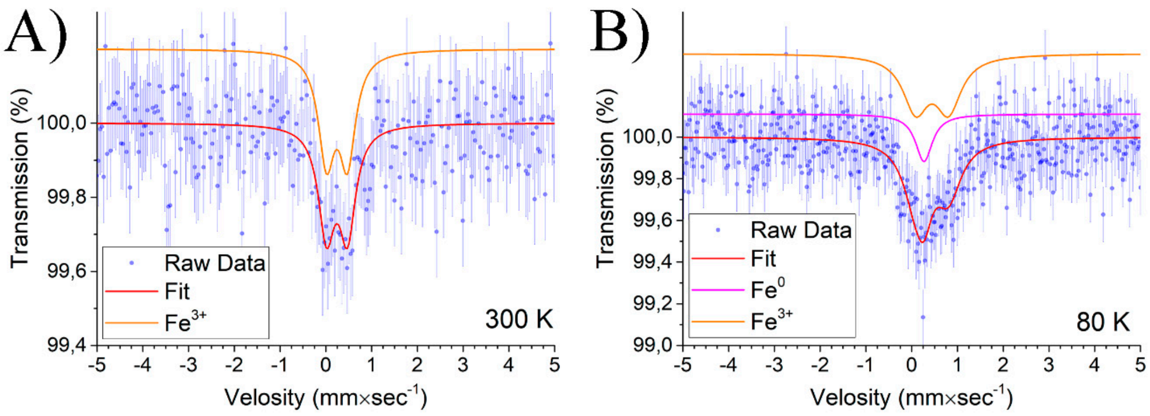

Figure S10 Mossbauer spectra of US-NP with deconvolution: A) at 300 K; B) at 80 K.

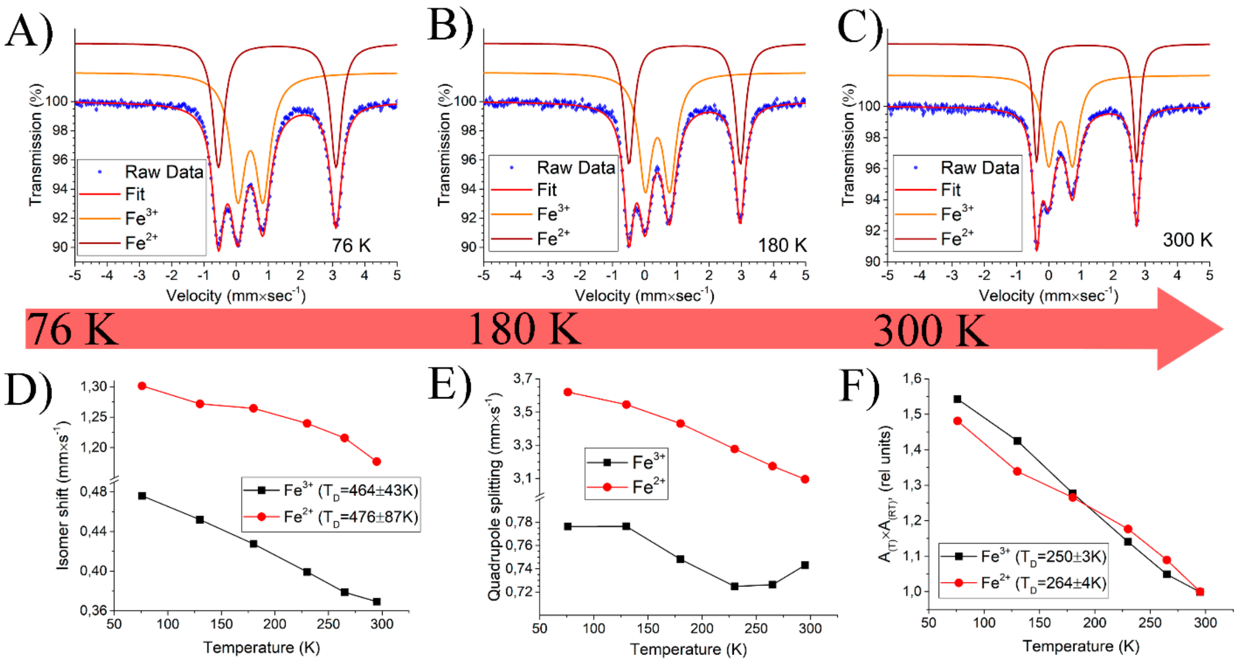

Figure S11 Mossbauer spectra of ChemRed-NP with deconvolution: A) at 76 K; B) at 180 K; C) at 300 K ; D) Isomer shifts at different temperatures; E) quadrupole splitting; F) Thermal deposition of ChemRed-NP

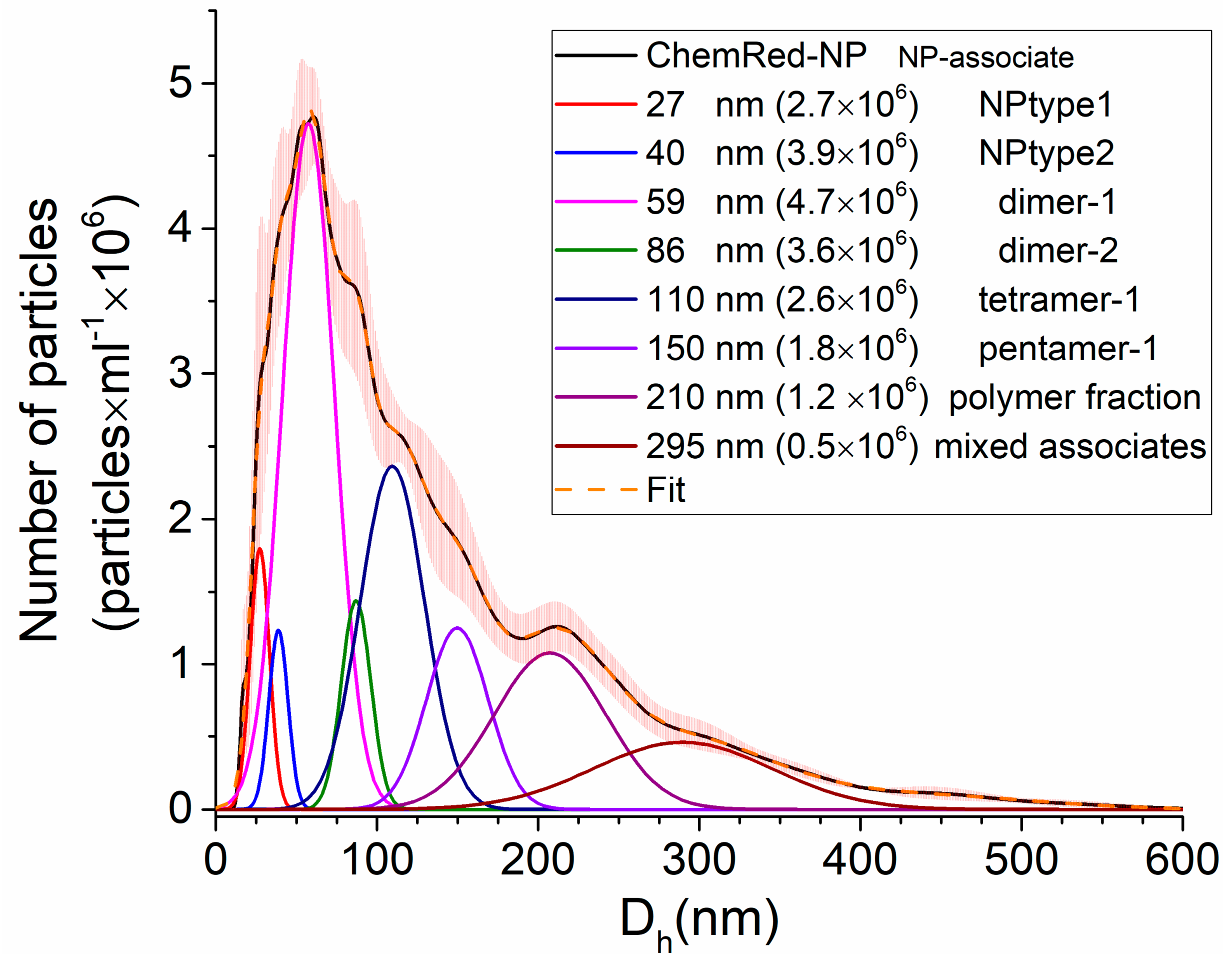

Figuere S12. NTA analysis ChemRed-NP

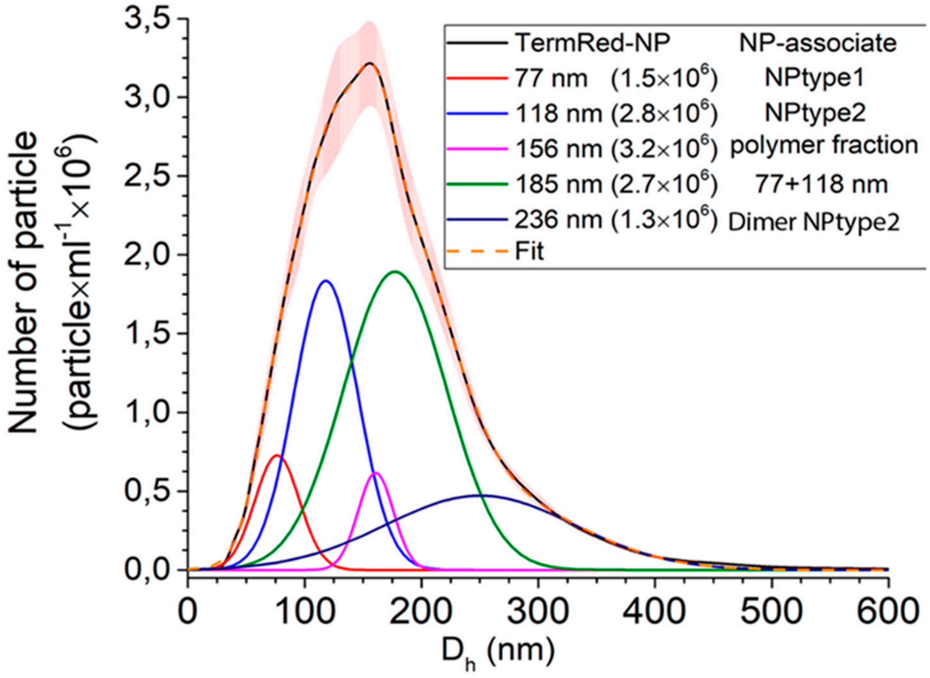

Figure S13. NTA analysis TermRed-NP

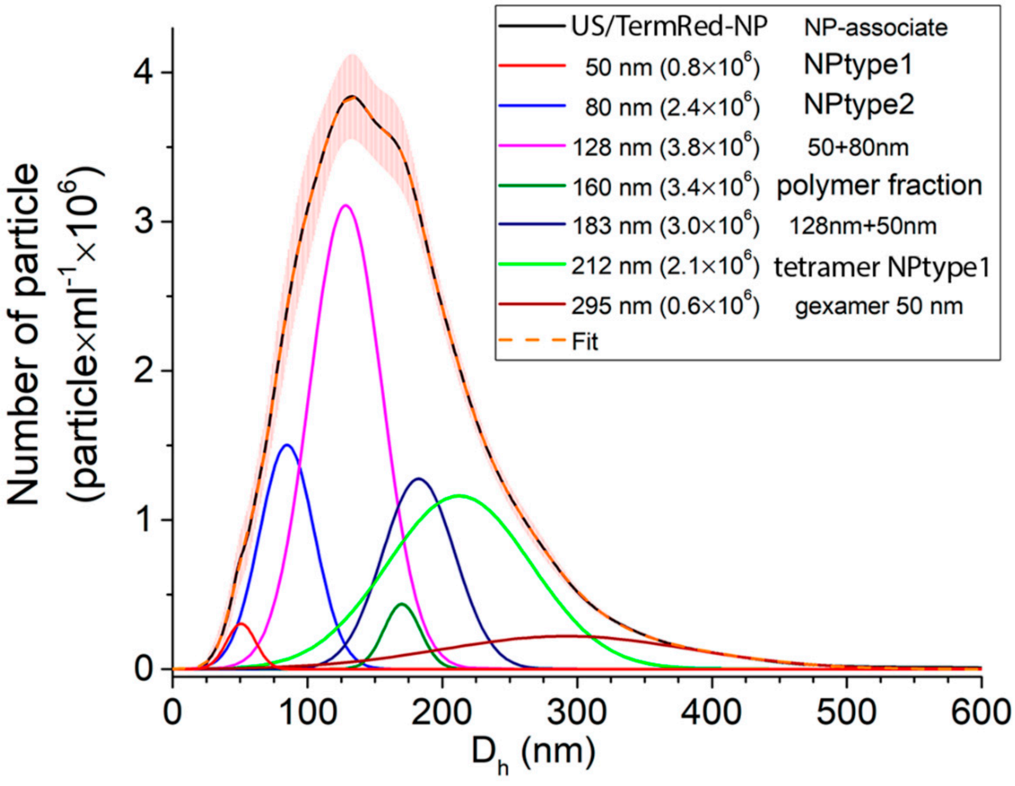

Figure S14. NTA analysis US/TermRed-NP

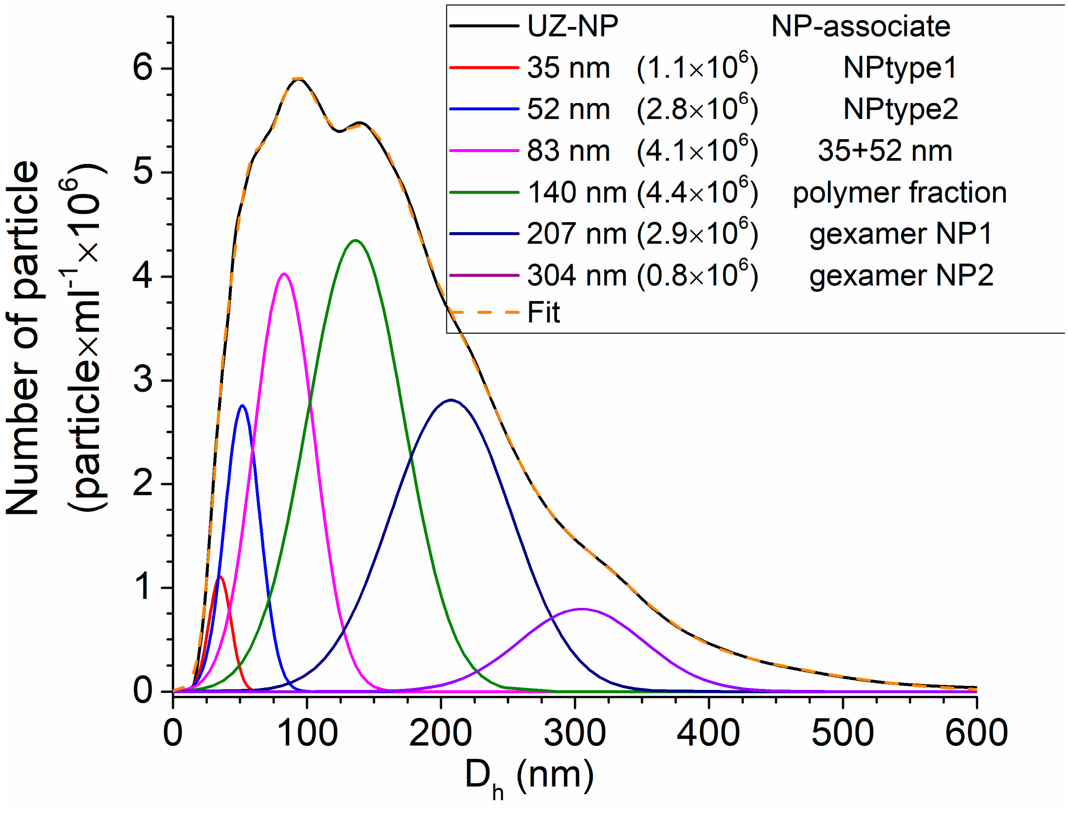

Figure S15. NTA analysis US-NP.
